# Cellular quantitative analysis of neuroblastoma tumor and splitting overlapping cells

**DOI:** 10.1186/1471-2105-15-272

**Published:** 2014-08-11

**Authors:** Siamak Tafavogh, Daniel R Catchpoole, Paul J Kennedy

**Affiliations:** Centre for Quantum Computation and Intelligent Systems (QCIS), Faculty of Engineering and IT, University of Technology, Sydney, PO Box 123, Broadway, NSW 2007 Sydney, Australia; Head of Biospecimens and Tumor Bank, Children’s Cancer Research Unit, The Kids Research Institute, The Children’s Hospital at Westmead, Locked Bag 400, Westmead, NSW 2145 Sydney, Australia; QCIS and the Head of School of Software in the Faculty of Engineering and IT, University of Technology, Sydney, PO Box 123, Broadway, NSW 2007 Sydney, Australia

**Keywords:** Splitting overlapping cells, Neuroblastoma tumor, Morphological analysis, Cell concave regions, Cell convex hull

## Abstract

**Background:**

Neuroblastoma Tumor (NT) is one of the most aggressive types of infant cancer. Essential to accurate diagnosis and prognosis is cellular quantitative analysis of the tumor. Counting enormous numbers of cells under an optical microscope is error-prone. There is therefore an urgent demand from pathologists for robust and automated cell counting systems. However, the main challenge in developing these systems is the inability of them to distinguish between overlapping cells and single cells, and to split the overlapping cells. We address this challenge in two stages by: 1) distinguishing overlapping cells from single cells using the morphological differences between them such as area, uniformity of diameters and cell concavity; and 2) splitting overlapping cells into single cells. We propose a novel approach by using the dominant concave regions of cells as markers to identify the overlap region. We then find the initial splitting points at the critical points of the concave regions by decomposing the concave regions into their components such as arcs, chords and edges, and the distance between the components is analyzed using the developed seed growing technique. Lastly, a shortest path determination approach is developed to determine the optimum splitting route between two candidate initial splitting points.

**Results:**

We compare the cell counting results of our system with those of a pathologist as the ground-truth. We also compare the system with three state-of-the-art methods, and the results of statistical tests show a significant improvement in the performance of our system compared to state-of-the-art methods. The F-measure obtained by our system is 88.70%. To evaluate the generalizability of our algorithm, we apply it to images of follicular lymphoma, which has similar histological regions to NT. Of the algorithms tested, our algorithm obtains the highest F-measure of 92.79%.

**Conclusion:**

We develop a novel overlapping cell splitting algorithm to enhance the cellular quantitative analysis of infant neuroblastoma. The performance of the proposed algorithm promises a reliable automated cell counting system for pathology laboratories. Moreover, the high performance obtained by our algorithm for images of follicular lymphoma demonstrates the generalization of the proposed algorithm for cancers with similar histological regions and histological structures.

## Background

Cancer is the common term for all malignant tumors, and Neuroblastoma is an infant cancerous tumor of the sympathetic nervous system [1]. Neuroblastoma Tumor (NT) is one of the most aggressive types of infant cancer and is the second most deadly cancer of infants. It has the lowest survival rate of all paediatric cancers [2]. One of the most important criteria in determining treatment for Neuroblastoma Tumors (NTs) is the quantitative analysis of neuroblast cells.

To determine a treatment for cancers, pathologists take a small tissue sample from the tumor, fix and stain it on a glass slide, then examine it under the microscope. Analyzing an enormous number of cells under the microscope is a tedious task, and the subject of inter- and intra-observer variability [3]. There is an urgent demand from pathologists for automated and robust systems to read the slides and perform cellular quantitative analysis [4].

The major difficulty in developing such automated systems is distinguishing between overlapping cells and single cells. This is becuase the histological slides are derived from 2-D sectioning of a 3-D tumor, which alters the morphology of cells. For example, two neighbouring cells in a 3-D space may appear as two overlapping cells in 2-D space. An enormous number of overlapping cell in the slides and the inability of the system to distinguish between different types of cells significantly reduces the accuracy of automated cell counting [5].

To count and split the overlapping cells, different algorithms and systems have been proposed [6]. However, we identified three main issues with those systems: 1) over-segmentation, 2) sensitivity to image quality, and 3) sensitivity to noise at the contour of the cells. For example, splitting algorithms based on the Watershed technique [7] are widely used in different systems [8–11], but intensive over-segmentation is a well known drawback of these techniques.

Intensity analysis approach is another type of algorithm for splitting the overlapping cells. Proposed systems in [12–15] follow the intensity analysis approach, and they identify splitting points based on higher intensity values in the overlap area. The main drawback of these methods is the sensitivity to the staining quality of tissue slides. For example, Hematoxylin and Eosin (H&E) is a staining method with low quality in revealing detail of cellular components. This reduces the performance of intensity based analysis methods in splitting overlapping cells, because these systems rely on the differences between the intensity of overlapping regions and non-overlapping regions.

Active contour method [16] is another approach that is used to analyze the contour of the overlapping cells. Active contour models are energy-minimizing curves which deform to fit image features. They determine the overlapping cells by detecting the regions that lie deeply inside the body of the cells. Active contour model developed by [17–19] detect and split overlapping cells, but the main drawback of these systems is their sensitivity to the false fluctuating and sharp corners at the cell contours.

Morphological-based analysis methods such as [20, 21] use the geometry of the cells in the images to find the cellular regions. The main problem with these systems is finding the correct markers of overlapping regions and neglecting the fluctuations in cells which falsely indicate overlapping regions. Many of the proposed systems consequently show sensitivity in detecting overlapping points.

Template Matching [22] is another technique for segmenting cells and splitting overlapping cells. Overlapping cells in tumor images are of different sizes and orientation, thus, the system neglects overlapping cells which have a high level of discrepancy from the defined template, as is the case with neuroblastoma.

To address the above issues, this paper introduces a novel automated cellular quantitative analysis in handling overlapping neuroblast cells. We propose new approaches and algorithms for identifying the overlapping regions based on the concave regions at the cell contours with low sensitivity to the image staining quality, and the false concave regions. We also develop an algorithm for splitting overlapping cells with low over-segmentation rate by associating the cell splitting operation to numbers of concave regions in overlapping cells. To develop the system, we first implement our previously proposed method [23] to distinguish overlapping cells from single cells using the differences in their morphological properties. We then develop novel overlapping cell splitting algorithms. Our system deploys dominant concave regions of the cells as markers to identify the overlap region. Dominant concave regions are identified by analyzing the convex-hull and area of the cells. The system then finds the initial splitting points at the critical points of the concave regions. To do this, the obtained concave regions are decomposed into their components such as arcs, chords and edges, and the distances between the components are analyzed using our seed growing technique. Finally, to determine the optimum splitting route between two candidate initial splitting points, we develop a shortest path determination approach. Moreover, the proposed algorithm has potential to be extended to other types of cancerous tumors with similar morphological characteristics in their cellular regions to NTs.

To test the system, we apply our algorithms to images of Hematoxylin & Eosin (H&E) stained slides. H&E is a widely used method for pathology labs [24] in which nuclei components are stained blue. We compare the results acquired by our system with those of a pathologist as the ground truth. We also evaluate our system by comparing it with state-of-the-art cell splitting systems such as the systems proposed by Kong et al. [21], Zhou et al. [25], and Fang et al. [26]. Moreover, the proposed algorithm has the potential to be extended to other types of cancerous tumors with similar morphological characteristics in their cellular regions to NTs such as follicular lymphoma. To validate the generalizability of our algorithm to the other types of cancer, we applied our algorithm and the above state-of-the-art algorithms to the H&E images of follicular lymphoma. Cellular regions within follicular lymphoma tumours have similar morphological characteristics to NTs such as dark blue and round nuclei with indiscernible cytoplasm. The results indicate that our proposed algorithm achieves high performance in splitting the overlapping cells for different datasets and cancer types.

## Methods

### Biological domain

Neuroblastoma tumors are embryonal malignancies of the sympathetic nervous system which originate from the neural crest. The prognosis of NT is related to several factors: 1) age of the patient, 2) location of the tumor, 3) surgical staging, and 4) microscopic grading [27]. The first three factors can usually be determined accurately and easily by pathologists; however determining microscopic grading requires extensive analysis of different histological regions and histological structures within tumor tissue under the microscope. Several microscopic grading schemes have been proposed for NT, among which the Shimada grading is the newest, most comprehensive, and widely used. According to the Shimada scheme, the level of tumor differentiation indicates the level of aggressiveness of the tumor. One of the most important criteria in determining the differentiation level is the number of neuroblast cells in the tumor tissue, which is the subject of this paper. A neuroblast cell is small to medium in size, with indiscernible to thin cytoplasm and vaguely defined cytoplasmic borders [27].

### Image acquisition, software and hardware

**Figure 1:**
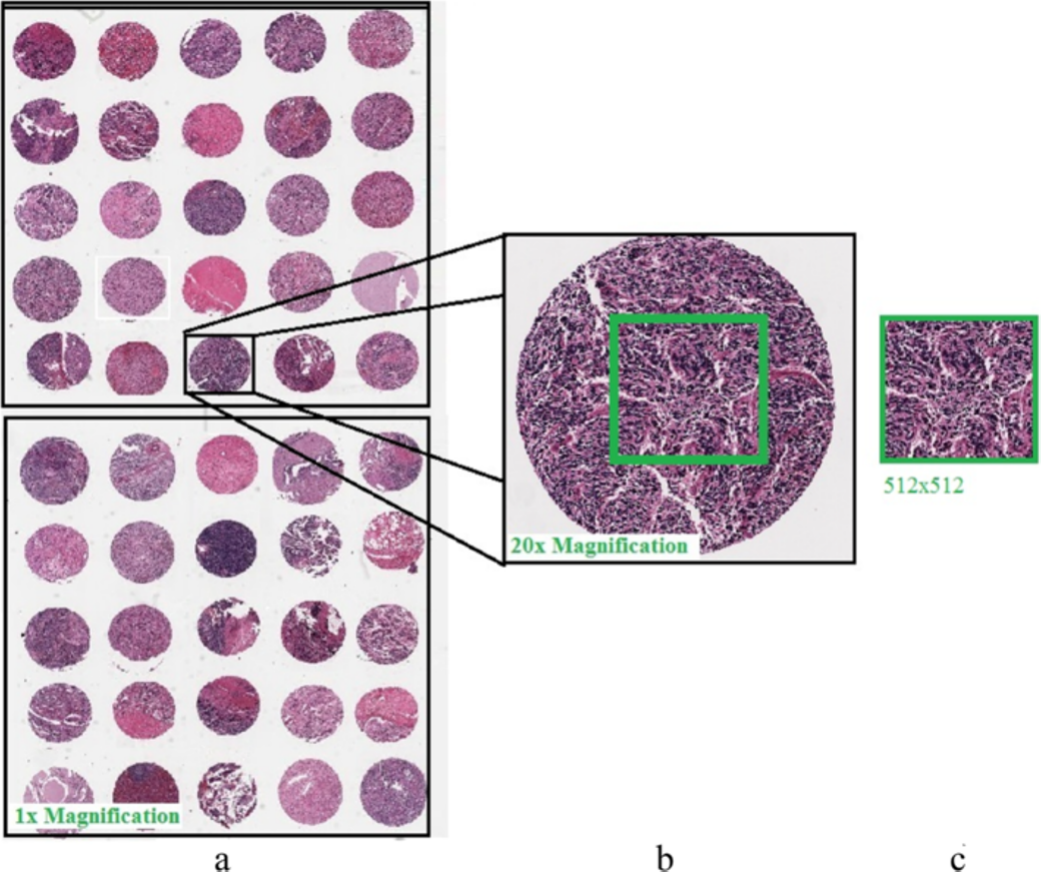
**A sample of tissue array. a)** an example of a tissue array taken from our dataset with 1× magnification, **b)** a random example of a tumor tissue with 20× magnification, and **c)** the cropped image of size 512×512.

To test and train our system, we used five NT datasets. We used one dataset for training and the other four datasets for testing the system. Each dataset is a digital tissue array containing 50 H&E stained tissue spots from different tumors at different neuroblastoma stages, as shown in Figure 1a. The images were scanned under 20 × magnification (standard magnification), in RGB color-space, as shown in Figure 1b. We cropped each of the tissue spots to an image of size 512×512 pixel regions with JPEG format and compression rate of 13:1, as demonstrated in Figure 1c. Thus each dataset contains 50 images of neuroblastoma samples from different tumors and in total our dataset contains 250 different images of NT tissue spots. The digital tissue arrays were provided by the Tumor Bank of the Kid’s Research Institute at the Children’s Hospital at Westmead (CHW), Sydney. The Tumor Bank is compliant with the local policy, national legislation and ethical mandates for the use of human tissue in research, in keeping with the Declaration of Helsinki. All samples were de-identified by the Tumor Bank. To demonstrate the generalizability of our developed algorithm, we also test our algorithm using histological images of follicular lymphoma which were deposited by [28] in a publicly accessible data repository at “http://web.mit.edu/huikong/www/projects.html” in the histopathological image analysis section. The dataset contains 21 H&E stained images of size 800×600 pixels which we cropped to size 512×512.

We developed our algorithm using MATLAB (The MAthWork, Inc, Natick, MA) and all experiments were run on a computer with 2 ×3.33 GHz CPU and 48GB of RAM.

### Segmentation of cellular regions

**Figure 2:**
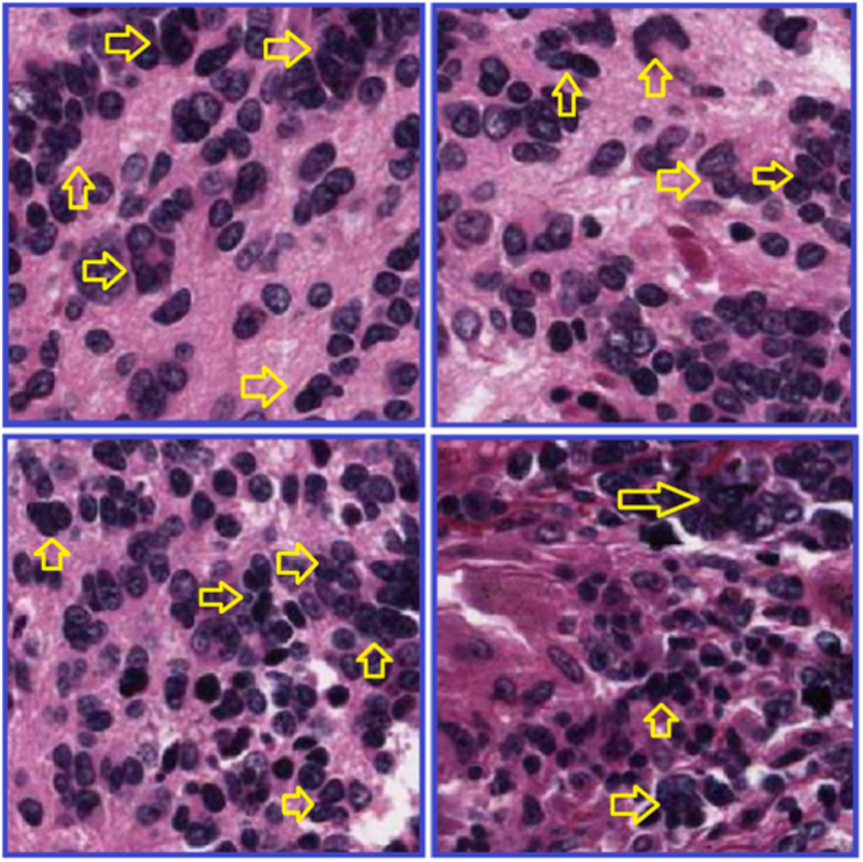
**Quality of H&E staining method.** Four different images from four different H&E stained tumor tissues. Yellow arrows indicate overlapping cells with unclear borders at the points of overlap.

**Figure 3:**
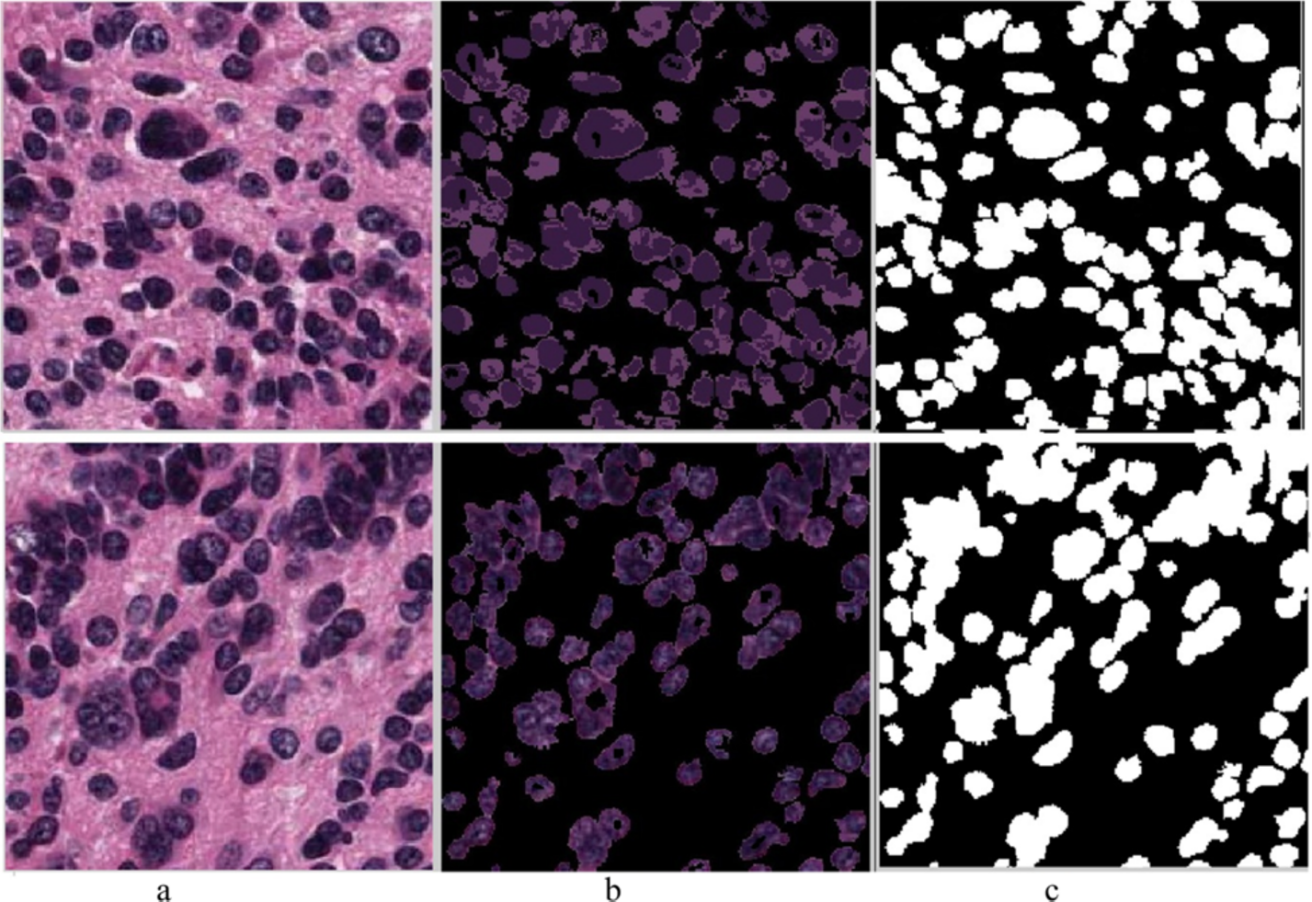
**The segmented cellular regions. a)** image of H&E stained tissue, **b)** segmented cellular regions, **c)** binary image of the segmented cellular region.

The main focus of this paper is the analysis of the cellular regions and splitting the overlapping cells, but prior to this taking place, the cellular regions must be segmented from other histological regions. The performance of the proposed algorithm for splitting overlapping cells depends on the quality of the cell segmentation. Low accuracy of cell segmentation reduces the performance of our proposed algorithm in distinguishing overlapping cells from single cells and splitting the overlapping cells. In this paper, we segment the cellular regions using our previously proposed hybrid algorithm [23]. The algorithm partitions images into many tiles and filters the constituent tiles of the cellular regions using a novel color analysis approach. The result is a foreground/background image, in which the foreground is made up of the constituent pixels of the cellular regions, and the background is 0. The intensity values of the cellular regions in the image of H&E stained tissues are not uniform and do not provide detailed information of the cellular components. The contours of most of the overlapping cells at the overlapping points are not clear because of the low quality of H&E staining as shown in Figure 2. This means that analyzing the H&E stained color images does not provide significant information for splitting overlapping cells, thus, to reduce the computational complexity and processing time, we transform the images of the segmented cells to binary images. The value of the pixels in the foreground of the binary images is 1 and in the background is 0. Figure 3 illustrates the original image, the image after segmentation, and the binary image.

### Algorithm description

**Figure 4:**
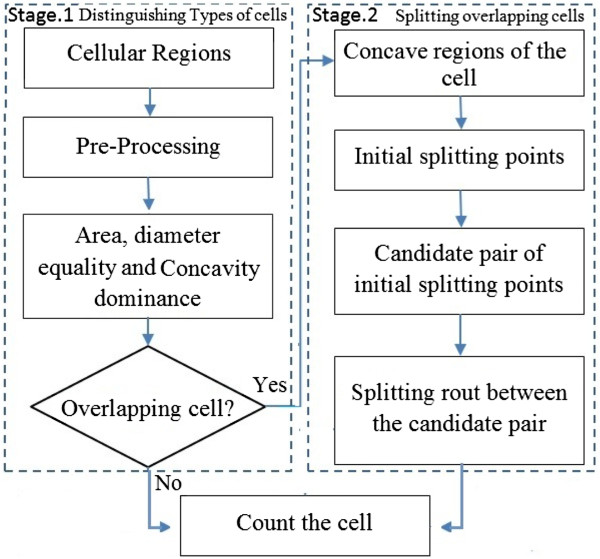
**Overview of our two stage algorithm.** In stage 1, different morphological properties of cells are analyzed for distinguishing between overlapping cells and single cells. In stage 2 the overlapping cells distinguished are split into single cells using our developed algorithm.

Our system analyzes the cells in two stages: 1) distinguishing overlapping cells from single cells, and 2) splitting overlapped cells into single cells. Stage 1 is addressed by applying the algorithm we developed in [23] which robustly discriminates the overlapping cells from the single cells. In stage 2, we develop a novel algorithm to split the overlapping cells distinguished in stage 1. Figure 4 gives an overview of our algorithms.

### Stage 1: Distinguishing types of cells

**Figure 5:**
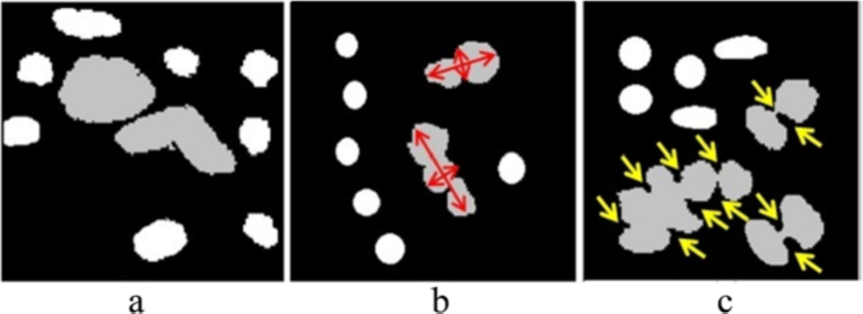
**Different morphology of overlapping cells and single cells.** The gray objects are overlapped cells, and the white objects are single cells. **a)** Bigger area for overlapped cells. **b)** Overlapped cells with unequal diameters. **c)** Higher concavity for overlapped cells.

To distinguish overlapping cells from single cells, pathologists consider certain morphological criteria of the cells such as size, diameter, and degree of cell concavity, as shown in Figure 5. In computerized analysis, the system does not comprehend the types of cell in the segmented cellular region, and thus considers both overlapping cells and single cells as the same object. We therefore develop an algorithm to assign one of the overlapping or single labels to each of the corresponding objects as follows.

#### Pre-processing

In the segmented cellular regions, cells might appear with holes or the segmented image might contain noisy artifacts, as shown in Figure 3b. Thus, prior to analyzing the segmented cells, a series of morphological operators, namely region filling, opening and closing operations are applied to the images. The size of the structuring element for the opening operation is 2, tuned empirically on 50 images from our training dataset. More details about the morphological operations can be found in [29]. These operations remove all the small holes within the body of cells and small noisy artifacts in the segmented image.

#### Area of the cells

The size of an overlapped cell is usually greater than the size of a single cell because an overlapped cell is formed by two or more cells. To distinguish overlapping cells from single cells, we must evaluate the area of each of the objects, and the average size of the objects in the image. The area of each cell is determined by counting the total number of constituent pixels within the cell, which is denoted as *A*_*i*_, ∀*i*∈{1,…,*N*} where *i* is the object number, and *N* is the total number of objects in the image. We then compute the average size of the objects in each image by .

#### Diameter equality

A single neuroblast cell is defined as having an approximately round shape [27]. This means that the major and minor diameters of a single cell are approximately equal in length. The major diameter is the longest straight line inside the cell and is the cell’s length. The minor diameter is a line perpendicular to the major diameter and is the cell width. We denote the ratio of the minor diameter to the major diameter of object *i* as *DE*_*i*_. If *DE*_*i*_ is bigger than a threshold *γ*, then object *i* is circular in shape. We obtain *γ*=0.9 empirically by tuning on 50 images from our training set. We analyze the performance of the algorithm using F-measure metric in discriminating overlapping cells from single cells.

#### Concavity dominance

The morphology of overlapping cells has greater concavity than a single cell. We use the following expression to calculate the concavity of the objects using,
1

where  is the area of convex hull for object *i*, and *CX*_*i*_ indicates the object concavity. Let us consider  as the average object concavity in the image.

#### Labeling the cells

**Figure 6:**
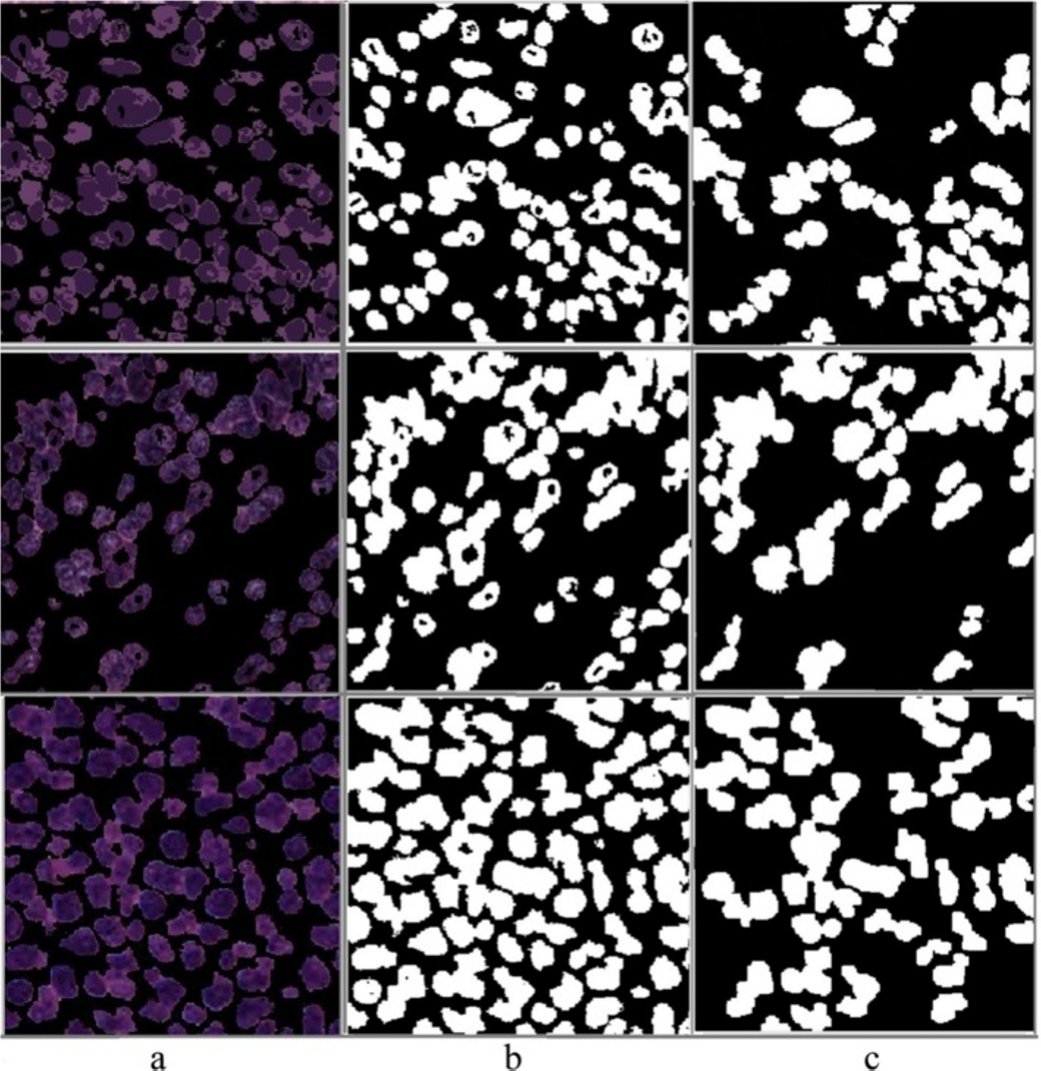
**The output of stage 1. a)** Indicates images of segmented cellular region with mixture of overlapping and single cells, **b)** demonstrates the binary images of the segmented cellular images without, and **c)** illustrates the segmented overlapping cells after applying the developed algorithm. Some of the artifacts and holes in b) do not exist in c), which is due to the applied preprocessing operations in stage 1.

To determine whether objects are overlapping or single, we assign a label to each of them. To do this, we first check the area of the cells using the following expression,
2

where PO and PS are Potentially Overlapping and Potentially Single labels respectively. In the next step, the ratio of minor to major diameters for each object is computed, and we assign new labels to the objects with respect to *ζ*_*i*_ using,
3

where SC is Single Cell label. The final step is to analyze the concavity of the objects with respect to  and change the labels as follows:
4

where OC is Overlapping Cell label. Ultimately, those objects with label  are considered as overlapping cells and the system stores them in a binary foreground/background image, where the foreground consists of the overlapping cells with a pixel value of 1, and the background is 0, as shown by Figure 6.

### Stage 2: splitting overlapped cells

The main markers that enable pathologists to identify the number of single cells in an overlapped cell are those areas which cut deep into the main body of the cell. These areas form concave shapes in the body of the cells. To decompose an overlapped cell into its constituent single cells, pathologists draw an imaginary line from one concave region to the counterpart concave region to split the overlapped cells.

To automate this process, our system first determines the concave regions of the cells. It then identifies initial splitting points on the concave regions, and selects the closest initial splitting points as the candidate pair of initial splitting points. Lastly, it determines the splitting routes using the shortest path between the selected candidate pairs.

#### Determining concave regions

**Figure 7:**
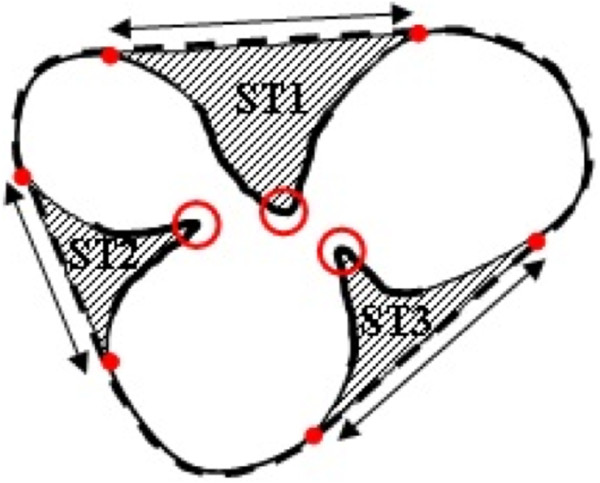
**An overlapping cell, and the morphological properties that are used in our splitting algorithm.** The dashed line indicates the convex hull, the white regions are the area of the cell, the shaded regions are *ST*s, the rings show the critical points on the arcs of the concave regions, the circles present critical points on the chords of *ST*s, and the arrows point to the chords of *ST*s.

To find the concave regions, we assume *R* and  are the entire region and the convex hull region of overlapping cells respectively. Then, we determine the regions between  and *R* using,
5

where *O* is the total number of overlapping cells that we obtained from stage 1. Most often these regions have similar morphology to triangles and we call those regions *Splitting Triangles* (*ST*s), as shown in Figure 7 by the hatched regions. Thus, *ϕ*_*j*_={*ST*_*k*_}, where *k*={1,…,*C*} and *C* is the total number of *ST*s for overlapping cell *j*. For example in this figure *C*=3 because the overlapping cell contains three *ST*s.

#### Initial splitting points

To split overlapping cells, we must first define the peak points of the regions which cut deep into the body of the cells as illustrated by the circles in Figure 7. We call the above-peak points the initial splitting points, because our algorithm initiates the splitting process from those points. These peak points are the critical points on the arcs of *ST*s. Thus, the first step is to determine the critical points of *ST*s.

For each *ST*, three critical points are identifiable: two critical points are on the chord of *ST* and are presented by circles and arrows in Figure 7, and one point is on the arc with maximum distance from the chord. To split the cells, we must consider the critical point on the arc only, and exclude the other two points from the analysis. To this end, we introduce an algorithm based on *ST* decomposition, seed growing techniques and critical point acquisition.

##### *ST*Decomposition

**Figure 8:**
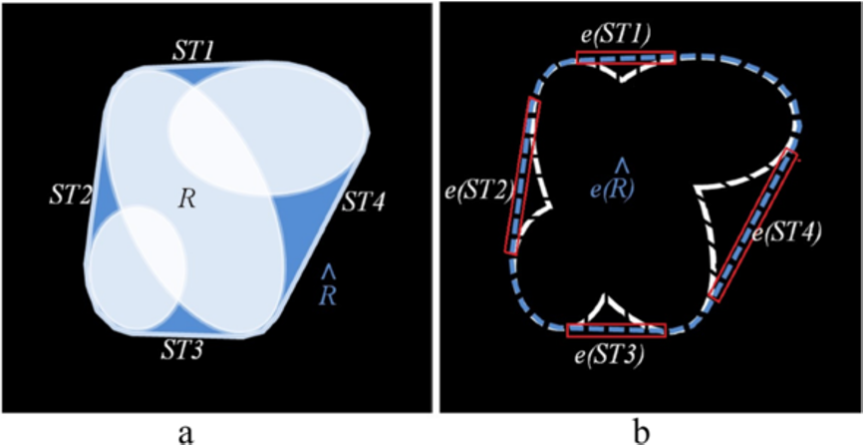
**Overlapping cell decomposition. a)** An example of three overlapping cells in which the white areas are the regions of the cells and the blue area is the convex hill of the overlapping cells. **b)** Illustrates the edge of the convex hull with blue dashed line, chords of STs with a red rectangle, and arcs with white dashed lines, which are lie deeply in the body of the cell.

We decompose each of the *ST*s into its edge, arc and chord. To determine the edge of the *ST*, Canny edge detection [30] is employed, and we denote the edge of *ST* by *e*(*ST*) as shown by the blue dashed line in Figure 8b. The next step is to find the chord of the *ST*. The chord is the only part of the *ST* which is tangent to the contour of the cell convex hull, as shown by the red rectangle in Figure 8b. To determine the chord, we therefore use the intersection between the edge of convex hull  of the cell and the edge of the *ST* using . Lastly, to identify the arc of *ST* as shown by the white dashed line in Figure 8b, we subtract the edge of *ST* from the chord by *arc*_*j*_(*ST*_*k*_)=*e*_*j*_(*ST*_*k*_)−*chord*_*j*_(*ST*_*k*_). The pixels on arcs and chords of all *ST*s are stored in two matrices **EA** and **EC** each with size *h*×*w* where *h* and *w* are equal to the height and the width of the original image respectively, which is 512×512 in our experiments. In each of the matrices, the pixels in the arc and the chord of overlapping cell *j* are set to *j*, and all other elements are set to 0.

##### Seed growing

**Figure 9:**
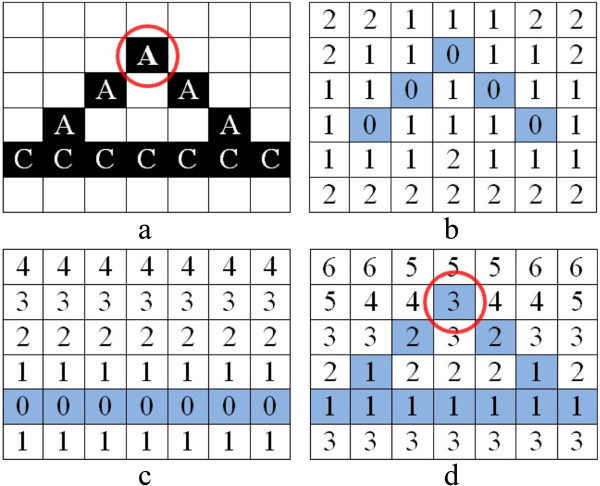
**Determining the critical point of a** ***ST*** **. a)** A and C represent the constituent pixels of an arc and a chord of an example *ST*, and the ring indicates the initial splitting point. **b)** Indicates **DA** where all zero elements in blue are seeds and all other elements are neighbors whose values are set based on the chessboard distance to their nearest seed. **c)** Illustrates **DC** with zero elements as seeds and neighbor elements. **d)** **DM**, which is obtained by adding the matrix in **b)** to the matrix in **c)**.

We develop the seed growing algorithm to find the initial splitting point of the identified *ST*s. The initial splitting point is located at the peak point of the arc with the maximum distance from the chord, as shown in Figure 9a by the circle. To identify the constituent pixels of an initial splitting point, we must compute the distance between pixels on the arc and the chord and our developed seed growing algorithm computes this distance. Note that the initial splitting point might be constructed by multiple pixels. This means that more than one pixel might be located at the peak point with the maximum distance from the chord. The algorithm creates two distance matrices **DA** and **DC** with size *h*×*w* based on matrices **EA** and **EC** for arcs and chords respectively. In these matrices those elements which have the same row and column numbers to the non-zero elements in **EA** and **EC** are considered 0, and we call them ‘seeds’, while all other elements are called ‘neighbors’. We assign the value of the neighbors by growing from the seeds to the neighbors. That is, the distance between each neighbor and its nearest seed is calculated by the chessboard distance, max(|*x*_*a*_−*x*_*b*_|,|*y*_*a*_−*y*_*b*_|) [31], where (*x*_*a*_,*y*_*a*_) and (*x*_*b*_,*y*_*b*_) are the coordinates of a neighbor element and seed element respectively. According to [32–34] the advantage of using chessboard distance over Euclidean distance in morphology analysis is that, whilst chessboard distance is computationally less expensive, it provides the same performance as Euclidean distance. Figures 9b and Figure 9c indicate matrices **DA** and **DC** with zero elements as the seeds and non-zero elements as the neighbors.

##### Critical point acquisition

The critical point of an arc is located at the maximum distance from the chord. We determine this using,
6

Those elements in **DM** having maximum values and aligned with the coordinates of the non-zero elements in **EA** are considered to be critical point of *ST*_*k*_, and the initial splitting points. Each *ST*_*k*_ in cell *j* has an initial splitting point. Figure 9d indicates **DM**.

#### Candidate pairs of initial splitting points

**Figure 10:**
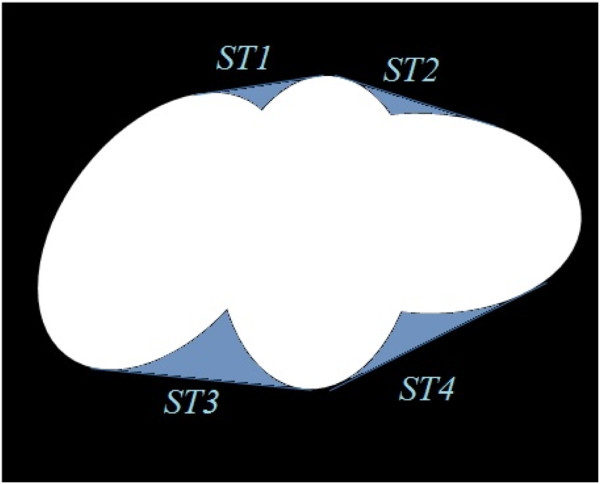
**Scenario 1.** An example of overlapping cells with four *ST*s, which are shown by gray regions.

**Figure 11:**
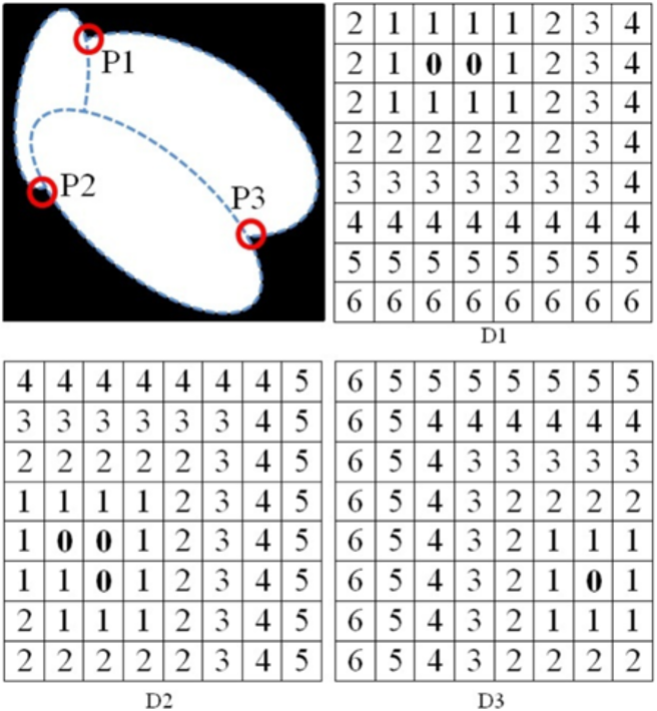
**Distance of each initial splitting point from the image borders.** An example of overlapping cell with three initial splitting points (*S* *P*=3), matrix distances *D*_1_ for *p*_1_, *D*_2_ for *p*_2_, and *D*_3_ for *p*_3_, and **0**s are the constituent pixels of P1, P2 and P3 in the cell. (The number of zeros in each of the matrices is different because the number of constituent pixels for each initial splitting point is different).

**Figure 12:**
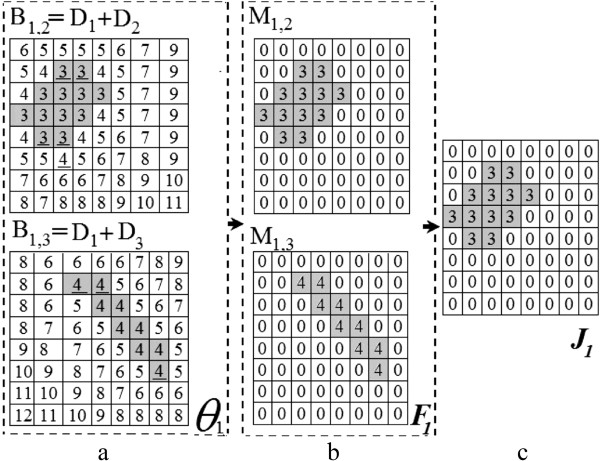
**Determining splitting pair by finding two closest critical points. a) D**_1_,**D**_2_ and **D**_3_ are from Figure 11, the underlined elements represent *p*_1_, *p*_2_ and *p*_3_, and the gray elements indicate the shortest distances between the two objects. **b)** **F**_1_ shows the shortest path between the objects, and **c)** **J**_1_ indicates that P1 and P2 in the example of Figure 11 are the closest objects.

We split an overlapping cell by connecting its nearest initial splitting points to each other. Thus, we must calculate the distance between the initial splitting points, and take two initial splitting points as a pair if they are located nearest to each other. We consider the two possible scenarios:

*Scenario 1: Overlapping cells with more than one ST*: Figure 10 indicates an overlapping cell with more than one *ST*. In this scenario, each of the obtained initial splitting points is considered as an object, although an initial splitting point in an image might consist of more than one pixel. Then for each object, we create a border distance matrix **D**_*p*_ with size *h*×*w*, where *p*={1,…,*SP*} and *SP* is the total number of objects (initial splitting points) for the overlapping cell *j*. We use our seed growing technique to set the elements of the matrix by considering the constituent pixels of the object as the seeds which are set to 0, and the value of the neighbors is set based on their chessboard distance to the nearest seed. Figure 11 is an example which indicates matrix **D**_*p*_ for each of the identified initial splitting points. To determine the shortest paths between two objects, the following four steps are developed:

1. For a selected object *p*, we introduce a set of matrices 7

where ***Θ***_*p*_ is a set of *SP*−1 matrices which indicates the distance between object *p* from the *SP*−1 other objects in an overlapping cell *j* as shown by the example in Figure 12a.

2. To obtain the shortest paths between object *p* and object *v*, we create a matrix **M**_*p*,*v*_ with size *h*×*w* in which all of its elements are 0 except those elements which have the same row and column numbers as the elements in **B**_*p*,*v*_ with minimum value.
3. **F**_*p*_ is a set of *SP*−1 matrices that have the smallest elements of the matrices in ***Θ***_*p*_ as illustrated in Figure 12b, 8
4. We determine the nearest object *v* to object *p* by finding **M**_*p*,*v*_ in set **F**_*p*_ which has the minimum value greater than zero among the other matrices. We store the results in **J**_*p*_ as shown in Figure 12c.

**Figure 13:**
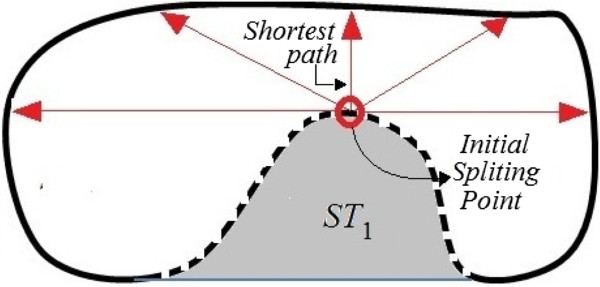
**Scenario 2.** An example of overlapping cells with one *ST*. The red arrows show the distance from the initial splitting point to the center of the overlapping cell.

*Scenario 2: Overlapping cells with one ST*: Figure 13 indicates an overlapping cell with one *ST*. Sometimes an overlapping cell might have only one concave region. In this case, the system considers that the other initial splitting point for making the splitting pair is among those pixels which are located at the contour of the cell closest to the first initial splitting point. For this, we develop an algorithm in two stages:

1. Determine the edge of the overlapping cells. Canny edge detection is used to acquire the edge of the overlapping cells, which we denote as *e*_*j*_(*R*) where *j*={1,…,*O*} and *O* is the total number of overlapping cells. We then use  to obtain all the edges of the overlapping cell except the edges which are in common to the concave region of the overlapping cell as shown by a solid line in Figure 13.

2. Identifying the shortest path between the initial splitting point of *ST*_1_ and the constituent pixels of *e*_*j*_(*R*)^′^ using the four steps from Scenario 1.

#### Splitting route determination

**J**_*p*_ contains all the possible shortest paths between two splitting points for overlapping cell *j*. To pick one shortest path from them, we consider all the pixels in the determined shortest path region as one object. Then, the thinning operation [35] is applied to the object for selecting a single shortest path. The thinning operation shrinks the object into a single line. The deployed thinning operation has two main stages [35]: 1) remove pixel *x* from the object if conditions 1, 2 and 3 are all satisfied; and 2) remove pixel *x* if conditions 1, 2 and 4 are all satisfied. Conditions 1 through 4, following [35] are given by

● **Condition 1:**

where


where *b*_*i*_ is obtained by


and *vx*_1_, *vx*_2_,…,*vx*_8_ are the values of the eight neighboring pixels of pixel *x*. We start from the east neighbor continuing counter clockwise.

● **Condition 2:**

● **Condition 3:**

● **Condition 4:**

**Figure 14:**
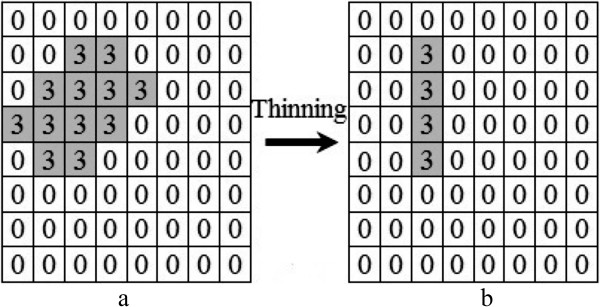
**Thinning operation. a)** is matrix **J**_1_ in Figure 12, and **b)** is *J* ^∗^_1_.

**Figure 15:**
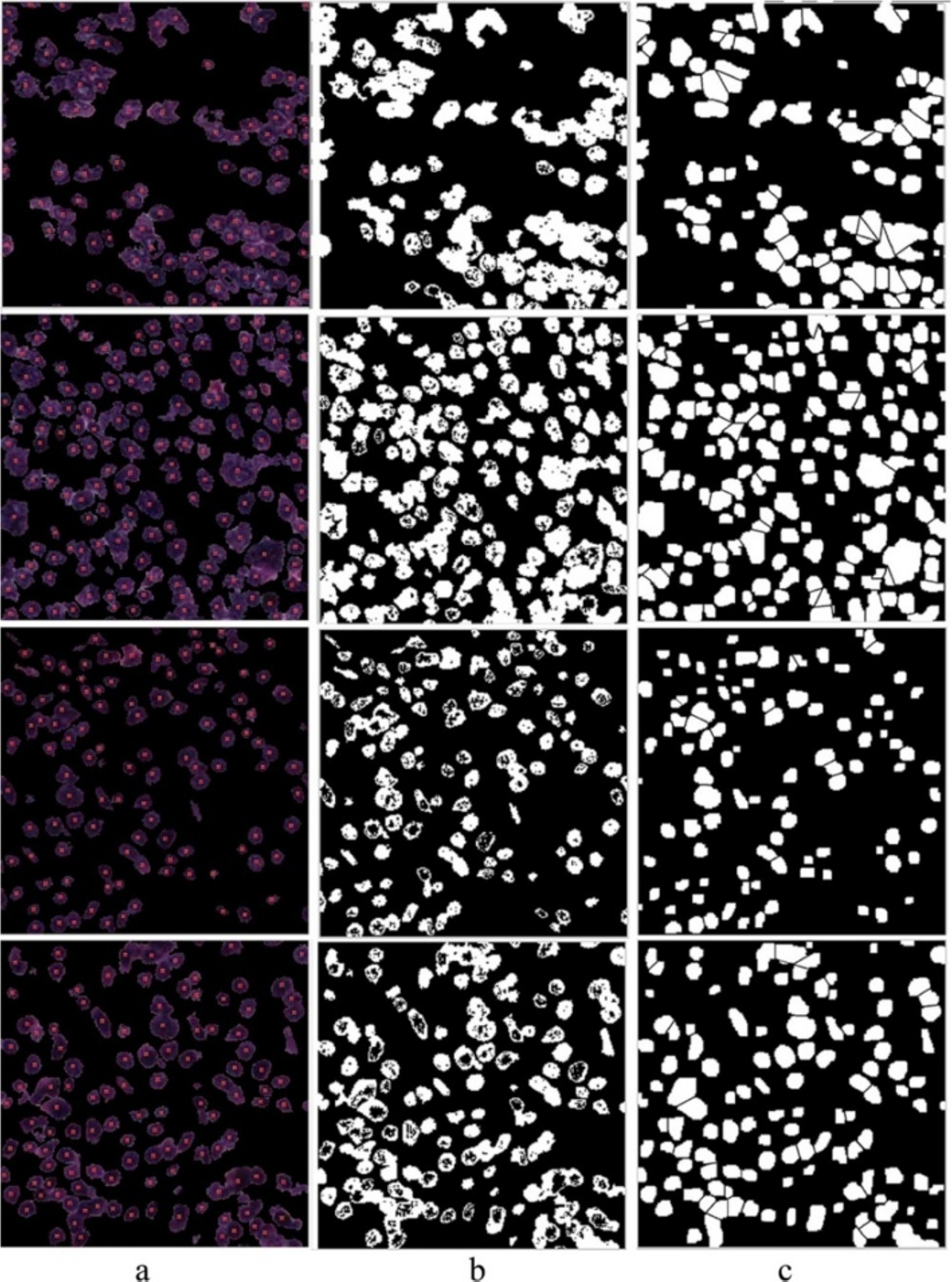
**Output of the developed algorithm for splitting overlapping cells.** **a)** shows the segmented cellular images that are used as the input for the algorithm, **b)** illustrates the binarized format of the segmented cellular regions, and **c)** indicates the output of the proposed algorithm.

We repeat the above two stage operations until we obtain an object with the width of a single pixel. The thinning algorithm always performs over the width of an object and it does not reduce the length of the object. The splitting points are always located at the longitudinal end points of an object. This means that the thinning operation automatically starts from one splitting point, reduces the width of the object and ends at the other splitting point. The results are stored in a matrix *J*^∗^ with size *h*×*w*. Figure 14 indicates **J**_*p*_ after the application of thinning operation. Lastly, we replace all the pixels in the original image with *x* and *y* coordinates equal to those non-zero elements in matrix  to 0. As a result, overlapping cells are split from their concave regions. Figure 15 shows an example of our algorithm outputs. The algorithm does not predict the initial shapes of the cells at the time of overlap, and it determines the number of constituent cells in a clump of overlapping cells only. This means that the system does not perform morphological restoration for the cells, and the system fragments the overlapping cells into the associated number of single cells. This is appropriate because the aim is to count the cells rather than to restore the morphology of the cells.

### Validation and comparisons

To validate the performance of our algorithm, we collaborated with a pathologist from CHW, Sydney as the ground truth. A recent audit of CHW shows that the historical misclassification for NTs in the Department of Histopathology from 1997 to 2012 is 0%, which guarantees the accuracy of our ground truth. We compare the cell counting results obtained by our system with those conducted manually by the pathologist.

A correctly segmented cell is a True Positive (TP). A cell that has been detected by our system but rejected by the pathologist is a False Positive (FP) or over-segmented, and a cell that has not been detected by our system but has been detected by the pathologist is a False Negative (FN) or under-segmented. To measure the performance of the system, we use precision, recall and F-measure [28]. We apply methods proposed by Kong et al. [21], Zhou et al. [25], and the Watershed technique [36] to the four test datasets to evaluate the performance of our system compared to other systems. The system proposed by Kong et al. splits the overlapping cells by smoothing the boundary of the cells using Fourier shape descriptors [37]. It splits overlapped cells from their dominant concave region by choosing a point *p*_*g*_ on the contour of the cell. It then finds *p*_*g*+*h*_ and *p*_*g*−*h*_ points on the cell contour which are *h*-points (*h* is set to 12) ahead of and behind of *p*_*g*_. According to Kong et al. [21], if more than 60% of the line  linking *p*_*g*+*h*_ and *p*_*g*−*h*_ is outside the overlapped cell, then *p*_*g*_ is considered to be a concave point [21]. The system splits the clump by cutting along the two detected concave points. This means that to split the overlapping cells, the system finds the concave regions by analyzing the contour of the cells and the angle between the line , while our system determines the convex hull and the peak point of the convex hulls. In the experiment, we show that relying on the contour of the cells increases the standard deviation and reduces the performance of the system in terms of splitting the overlapping cells. The system proposed by Zhou et al. applies the Watershed transform to the overlapped cells, and uses a novel hybrid merging algorithm to reduce the negative effects of high over-segmentation derived by Watershed technique. This algorithm combines the compactness score with the probability distribution function score to merge the fragmented cells produced by the Watershed technique. Watershed technique finds seeds of the objects by identifying the innermost regions of the objects. Each of the seeds receives a unique identification label and the area around each of the seeds is grown. The pixels within the area receive an identical label as their corresponding seed. The area around each seed grows until it meets an area around another seed with a different identification label. Those pixels at the collision points form the Watershed line. Qi et al. [13] developed a two step system for identifying and splitting the overlapping cells. In the first step, the system determines the geometric center of each cell by integrating a shifted Gaussian kernel with mean shift algorithm [38]. The shifted Gaussian kernel gives the highest votes to the pixels located at the maximum distance from the contour of the cell. This means that the pixels located at the center of the cell receive the maximum vote. For the overlapping cells, the shifted Gaussian kernel considers the overlapping regions as those with the higher gradient intensity and gives higher votes to the regions outside the overlapping regions. For example, if two cells overlap, the pixels on both sides of the overlapping region receive the higher votes. This means that the algorithm finds the seeds of all the constituent cells of an overlapping cell. The system then applies mean shift algorithm to the constituent pixels of the identified seeds to calculate the center of cells and discriminates the overlapping cells from single cells. The seeds obtained in stage 1 are used to detect the initial position of the segmentation of the overlapping cells. In the second step, the algorithm extracts the contour of the cells using a level set function to detect the overlapping cells.

## Results and discussion

Each row of Table 1 indicates the average TP, FP, FN, precision, recall and F-measure of our system for each dataset after comparison with the results of the pathologist. Table 2 indicates that our system has the best average F-measure, followed by Kong et al. with an average F-measure of 84.30%, which is the best result of the state-of-the-art methods, but which is approximately 4% lower than the F-measure obtained by our system. The system proposed by Qi et al. obtains the lowest F-measure of 83.21%.

**Table 1:** **The results obtained by our system**

Data	TP	FP	FN	Precision	Recall	F-measure
Dataset.1	106.20	14.95	11.55	87.66%	91.00%	89.29%
Dataset.2	146.83	23.57	17.19	86.16%	89.52%	87.80%
Dataset.3	181.98	25.11	23.31	87.87%	88.64%	88.25%
Dataset.4	105.64	12.02	12.91	89.78%	89.11%	89.44%
Average				87.86%	89.56%	88.70%
StdDev				1.48%	1.02%	0.79%

**Table 2:** **The average F-measure obtained by state-of-the-art systems**

Data	Our System	Kong et al.	Zhou et al.	Qi et al.
Dataset.1	89.29%	83.38%	82.75%	82.58%
Dataset.2	87.80%	85.37%	84.68%	83.99%
Dataset.3	88.25%	82.32%	82.28%	83.77%
Dataset.4	89.44%	86.13%	83.72%	82.50%
Overall	88.70	84.30%	83.36%	76.66%
StdDev	0.79	1.75	1.07	0.78

According to Table 2, the system proposed by Kong et al. has the highest standard deviation. The main reason for this is that the system relies on cell contour for splitting overlapping cells. Tables 3 and 4 indicate that our system obtains the best precision and recall, while the system proposed by Qi et al. achieves the lowest precision and recall among the state-of-the-art methods. The proposed system by Zhou et al. addresses the over-segmentation drawback of the watershed technique by merging the fragmented cells. Although the system developed by Zhou et al. improves the performance of the watershed technique, the lower precision results compared to Kong et al. and our system is an indication of the higher over-segmentation rate for the system proposed by Zhou et al.

**Table 3:** **The average precision obtained by state-of-the-art systems**

Data	Our System	Kong et al.	Zhou et al.	Qi et al.
Dataset.1	87.66%	82.67%	81.46%	81.56%
Dataset.2	86.16%	84.08%	84.61%	82.91%
Dataset.3	87.87%	82.19%	80.12%	83.16%
Dataset.4	89.78%	84.51%	82.41%	80.19%
Overall	87.86%	83.36%	82.15%	81.96%
StdDev	1.48	1.10	1.88	1.37

**Table 4:** **The average recall obtained by state-of-the-art systems**

Data	Our System	Kong et al.	Zhou et al.	Qi et al.
Dataset.1	91.00%	84.11%	84.08%	83.62%
Dataset.2	89.52%	86.71%	84.77%	85.10%
Dataset.3	88.64%	82.46%	84.57%	84.39%
Dataset.4	89.11%	87.83%	85.08%	84.95%
Overall	89.56%	85.27%	84.62%	84.52%
StdDev	1.02	2.44	0.42	0.67

Lastly Table 5 provides the results of the Holm statistical tests [39]. Holm is used for rigorous statistical tests [40] and is a well-known method in reporting biological results with multiple hypotheses [41]. We choose Holm test to be consistent with the approaches which are used by the health researchers in reporting their statistical results. The null hypothesis of the tests is given as the similarity between the precision, recall and F-measure of our proposed algorithm and those of the algorithm proposed by Kong et al. The statistical test indicates that the improvement in performance of our system compared to the above state-of-the-art algorithms is statistically significant. The algorithm proposed by Kong et al. is chosen for the statistical tests, because it obtained the best performance among other state-of-the-art algorithms in Tables 2, 3 and 4. Table 5 shows that *p*-values for precision, recall and F-measure for each of the four test datasets are less than the level of significance (*α*=0.1), and consequently the hypothesis is rejected. These results conclude that the improvement in performance of our algorithm compared to the algorithm proposed by Kong et al. is statistically significant.

**Table 5:** **Holm statistical tests for precision, recall and F-measure of the algorithm proposed in this thesis and the algorithm proposed by Kong et al. in splitting overlapping cells with** ***α*** **=0** ***.*** **1 and the obtained standard scores (** ***Z***_**0**_ **)**

Dataset	Metric	***z***_0_	***p***-value
	Precision	4.024	5.699 ×10^−5^
**Dataset 1**	Recall	4.024	5.699 ×10^−5^
	F-measure	3.13	0.001
	Precision	2.683	0.007
**Dataset 2**	Recall	2.459	0.001
	F-measure	3.13	0.001
	Precision	4.248	2.151 ×10^−5^
**Dataset 3**	Recall	3.801	1.439 ×10^−4^
	F-measure	3.801	1.439 ×10^−4^
	Precision	4.248	2.151 ×10^−5^
**Dataset 4**	Recall	3.130	0.01
	F-measure	2.459	0.01

### Algorithm generalizability

To demonstrate the generalizability of our algorithm for new datasets with different types of cancer, we evaluate our algorithm with the follicular lymphoma dataset that was used by [21]. Table 6 indicates the F-measure of our algorithm and the state-of-the-art algorithms. The results show that our algorithm obtains the highest performance in this dataset with an average F-measure of 92.79% compared to the other algorithms. The algorithm proposed by Kong et al. obtains the best performance among other state-of-the-art algorithms but its performance is approximately 1% lower than the F-measure obtained by our algorithm. The main reason for the higher F-measures obtained by all of the algorithms in the follicular lymphoma dataset compared to the NT dataset is the higher contrast between the different types of histological region as a result of the better image quality.

**Table 6:** **The average F-measure and precision and recall for our system and state-of-the-art system by applying them to the follicular cancer dataset provided by Kong et al**

Algorithms	Avg(F-measure)	Avg(Precision)	Avg(Recall)
Our System	92.79%	93.38%	92.21%
Kong et al.	91.70%	92.04%	91.37%
Zhou et al.	88.46%	88.02%	88.91%
Watershed	81.11%	81.83%	80.41%

## Conclusion

To address the issue of overlapping cells in cellular quantitative analysis, this paper has proposed a novel system in two stages: 1) distinguishing overlapping cells from single cells based on their morphological differences, and 2) splitting overlapping cells into single cells using our splitting triangle decomposition, seed growing and shortest path determination techniques. To measure the accuracy, we compared the results of our system with those of a pathologist as a ground truth using F-measure. We also evaluated the system by comparing with systems proposed by Kong et al. [21], Zhou et al. [25], and the Watershed technique [42] using histological images derived from different tissue arrays and different types of cancer. The highest precision and recall obtained by our system compared to these systems show the lowest over-segmentation and under-segmentation respectively for our system. The system proposed by Qi et al. obtains the lowest F-measure, precision and recall compared to the other methods tested. The system is an intensity-based method which discriminates overlapping cells from single cells by relying on the higher grading intensity of the overlapping region, while the H&E staining methods in most of the cases is incapable of revealing those details. The system proposed by Zhou et al. significantly reduces the over-segmentation drawback of the Watershed-based by almost 8% in neuroblastoma and more than 7% in follicular lymphoma datasets, but the low overall F-measure of the system compared to ours and that of Kong et al. [21] indicates, that Watershed-based techniques, in comparison to those based on morphological analysis, do not provide optimum performance in splitting the overlapping cells. Moreover, the standard deviation obtained by our system was the lowest compared to other systems. The Watershed technique and the method proposed by Kong et al. obtains the highest standard deviation which demonstrates their fluctuating performance on different datasets. Our system cannot accurately identify and split highly overlapped cells with very low numbers of convexities on their contours. Discriminating and splitting those cells is a challenging task which even pathologists, as a gold standard, cannot manage. This paper proposes a novel overlapping cell splitting approach by introducing a two step algorithm which distinguishes overlapping cells from single cells by exploiting their morphological differences, and splitting those overlapping cells by proposing an approach which determines the initial splitting points at the critical points of the concave regions of overlapping cells. The algorithm proposed in this paper obtains the highest performance in quantitative analysis of cellular regions within NTs among the tested state-of-the-art algorithms. The high accuracy obtained by our algorithm in cell counting of NTs enhances the process of making prognosis for pathology laboratories, and reduces the inter- and intra- observer variability. Moreover, the high performance obtained by our algorithm in the quantitative analysis of the images of follicular lymphoma guarantees the robustness and generalizability of the proposed algorithm in the quantitative analysis of cellular regions with similar morphological characteristics to NTs.

## Authors’ information

ST has research expertise in medical image processing and in designing computer aided diagnosis systems, and this project was part of his ST PhD program. ST currently works as a postdoctoral research on a grant provided by Cancer Institute NSW. DC throughout his career has focused on the molecular basis of paediatric malignancies. His scientific achievements and publications have concentrated on the assessment of childhood tumors with specific attention given to Neuroblastoma and Leukaemia. PK has research expertise in health and medical data analytics. He has huge practical data analytic and software development experience both in industry since 1985, and in academic projects since 1999. He is Director of the Knowledge Infrastructure Laboratory (KIL) in the Centre for Quantum Computation and Intelligent Systems.
